# Quantifying the activity profile of ASO and siRNA conjugates in glioblastoma xenograft tumors *in vivo*

**DOI:** 10.1093/nar/gkae260

**Published:** 2024-04-13

**Authors:** Samantha L Sarli, Hassan H Fakih, Karen Kelly, Gitali Devi, Julia M Rembetsy-Brown, Holly R McEachern, Chantal M Ferguson, Dimas Echeverria, Jonathan Lee, Jacquelyn Sousa, Hanadi F Sleiman, Anastasia Khvorova, Jonathan K Watts

**Affiliations:** RNA Therapeutics Institute, University of Massachusetts Chan Medical School, Worcester, MA, USA; RNA Therapeutics Institute, University of Massachusetts Chan Medical School, Worcester, MA, USA; RNA Therapeutics Institute, University of Massachusetts Chan Medical School, Worcester, MA, USA; RNA Therapeutics Institute, University of Massachusetts Chan Medical School, Worcester, MA, USA; RNA Therapeutics Institute, University of Massachusetts Chan Medical School, Worcester, MA, USA; RNA Therapeutics Institute, University of Massachusetts Chan Medical School, Worcester, MA, USA; RNA Therapeutics Institute, University of Massachusetts Chan Medical School, Worcester, MA, USA; RNA Therapeutics Institute, University of Massachusetts Chan Medical School, Worcester, MA, USA; RNA Therapeutics Institute, University of Massachusetts Chan Medical School, Worcester, MA, USA; RNA Therapeutics Institute, University of Massachusetts Chan Medical School, Worcester, MA, USA; Department of Chemistry, McGill University, Montréal, Québec, Canada; RNA Therapeutics Institute, University of Massachusetts Chan Medical School, Worcester, MA, USA; Program in Molecular Medicine, University of Massachusetts Chan Medical School, Worcester, MA, USA; RNA Therapeutics Institute, University of Massachusetts Chan Medical School, Worcester, MA, USA; Department of Biochemistry and Molecular Biotechnology, University of Massachusetts Chan Medical School, Worcester, MA, USA

## Abstract

Glioblastoma multiforme is a universally lethal brain tumor that largely resists current surgical and drug interventions. Despite important advancements in understanding GBM biology, the invasiveness and heterogeneity of these tumors has made it challenging to develop effective therapies. Therapeutic oligonucleotides—antisense oligonucleotides and small-interfering RNAs—are chemically modified nucleic acids that can silence gene expression in the brain. However, activity of these oligonucleotides in brain tumors remains inadequately characterized. In this study, we developed a quantitative method to differentiate oligonucleotide-induced gene silencing in orthotopic GBM xenografts from gene silencing in normal brain tissue, and used this method to test the differential silencing activity of a chemically diverse panel of oligonucleotides. We show that oligonucleotides chemically optimized for pharmacological activity in normal brain tissue do not show consistent activity in GBM xenografts. We then survey multiple advanced oligonucleotide chemistries for their activity in GBM xenografts. Attaching lipid conjugates to oligonucleotides improves silencing in GBM cells across several different lipid classes. Highly hydrophobic lipid conjugates cholesterol and docosanoic acid enhance silencing but at the cost of higher neurotoxicity. Moderately hydrophobic, unsaturated fatty acid and amphiphilic lipid conjugates still improve activity without compromising safety. These oligonucleotide conjugates show promise for treating glioblastoma.

## Graphical Abstract

**Graphical Abstract:**
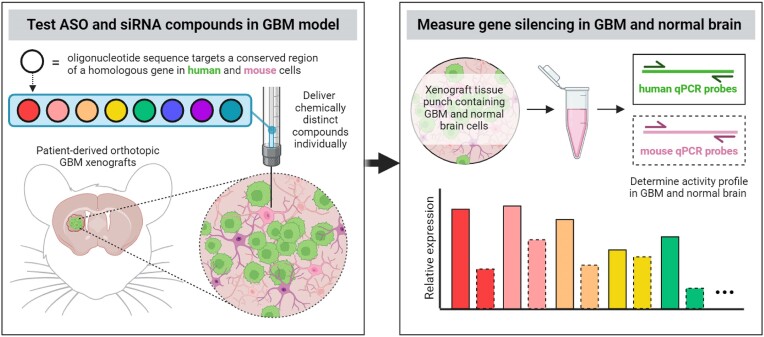

## Introduction

Glioblastoma multiforme (GBM) is the most common high-grade malignant brain tumor in adults with an average survival time of 12–15 months after diagnosis (1,2). Treatment consists of maximal tumor resection, followed by radiation and chemotherapy with the drug temozolomide (TMZ). However, efficacy of these standard as well as experimental therapies are limited by these tumors’ extensive infiltration into adjacent brain tissue and high intratumoral heterogeneity (3,4). As a result, patient prognosis has not improved in over two decades and effective treatment options remain a major unmet clinical need. Novel therapies that overcome GBM-intrinsic barriers are essential for improving patient outcomes.

Therapeutic oligonucleotides are chemically modified nucleic acid drugs that can silence disease relevant transcripts in a sequence-dependent manner. There are two mechanistically distinct classes of gene-silencing oligonucleotides: antisense oligonucleotides (ASOs) and small-interfering RNAs (siRNAs) (5). As programmable, targeted therapies, both ASOs and siRNAs can potently and specifically silence virtually any oncogene driving GBM tumor growth and progression. Indeed, several oligonucleotide compounds have already been tested in clinical trials for GBM patients (trabedersen, aprinocarsen, and imetelstat) (6–8). Despite being programmed to inhibit therapeutically relevant targets (TGFβ, PKCα and telomerase, respectively), these oligonucleotides largely featured chemistry that was neither pharmacologically optimized for the brain nor brain tumors.

All ASOs and siRNAs require chemical modification for sufficient functional activity *in vivo*. The type and position of chemical modifications dramatically influences the pharmacology of therapeutic oligonucleotides. Oligonucleotides being developed for neurological diseases incorporate chemical designs that support robust tissue distribution in the central nervous system (CNS), durable (multi-month) gene silencing, and low neurotoxicity and immune activation (9). Malignant GBM tumor cells are molecularly distinct from normal brain cells and it is unknown whether ASO and siRNA chemical designs that are optimized for activity in the CNS will also achieve efficacious gene silencing in brain tumors.

In this work, we directly measure ASO- and siRNA-mediated gene silencing in normal brain and brain tumor cells. To do this, we used an ASO or siRNA sequence which targets homologous sequences in mRNA transcripts produced by both the tumor and normal brain cells in patient-derived orthotopic GBM xenografts. Using species-specific qPCR probes we can then quantify and differentiate the extent of mRNA silencing in (human) tumor cells and (mouse) normal brain cells collected from a single tumor xenograft biopsy. The fact these GBM xenografts are invasive means that punches near the site of tumor implantation consistently include both human and mouse cells in abundance. We applied this assay to two different mRNA targets to study the silencing ability of ASOs and siRNAs including five types of conjugates. We observed that the most consistent gene silencing activity in GBM cells after intratumoral or intracerebroventricular injection was exerted by siRNAs conjugated to moderately hydrophobic, unsaturated fatty acids and amphiphilic lipids.

## Materials and methods

### Synthesis of oligonucleotides

Unconjugated ASOs were synthesized on a Dr Oligo 48 synthesizer. 2′-*O*-methoxyethyl (MOE)-modified phosphoramidites were coupled for 8 min. ASOs were deprotected in concentrated aqueous ammonia (30% in water) at 55 °C for 16 h and characterized by liquid chromatography–mass spectrometry (LC–MS). Final desalting was effected by diafiltration (3× water wash) in a 3-kDa cutoff Amicon centrifugal filter.

For folate-conjugated ASOs, a constrained alkyne functionality, bicyclo[6.1.0]non-4-yne (BCN), was conjugated at the 5′-end of *HTT/Htt* 5–10-5 MOE-DNA gapmer sequence during solid phase synthesis using a Peg2-BCN-phosphoramidite. A (dT)_5_ linker was used between the actual *HTT/Htt* gapmer sequence and the BCN functionality to ensure cleavability of the folate moiety to be embedded in the following step. The resulting oligonucleotide was deprotected using standard 30% ammonium hydroxide deprotection method and desalted using Amicon filters. The identity and integrity of the oligonucleotide was confirmed by LC–MS. A stock solution of 25 mM Folate-Peg2-N_3_ (purchased from Axispharm) was prepared in RNase-free water. Folate-Peg2-N_3_ (100 uM, 1eq) and BCN conjugated *HTT/Htt* sequence (1000 uM, 1eq) were mixed in water and allowed to react for 30 min at room temperature. Unreacted small molecules and salts in the reaction mixture were removed by Amicon desalting and MW of the resulting folate conjugated *HTT/Htt* sequence was confirmed by LC–MS.

For siRNAs, oligonucleotides were synthesized on a MerMade 6/12 synthesizer (Bioautomation) following standard protocols. Conjugated and di-branched sense strands were synthesized at 5–20 μmol scales on custom-synthesized lipid- or branchpoint-functionalized controlled pore glass (CPG) supports for DCA, EPA, and divalent oligonucleotides as previously described (10–12). For the amphiphilic dendrimer-conjugated sense strand, synthesis was on a CPG functionalized with UnyLinker (ChemGenes) and commercially available amidites (i.e. C6, C12 and symmetrical branching amidite from ChemGenes and Glen Research) were used to build the dendritic moiety on the 5′-end as previously described (13). All sense strands had a dT_2_ spacer between the oligonucleotide and the conjugate. All strands were cleaved and deprotected using 28% aqueous ammonium hydroxide solution for 20 h at 55°C, followed by drying under vacuum at 40°C, and resuspension in Millipore H_2_O. Oligonucleotides were purified using an Agilent Prostar System (Agilent Technologies) on a C18 column for lipid-conjugated sense strands and an ion-exchange column for antisense strands. Purified oligonucleotides were desalted by size-exclusion chromatography and characterized by LC-MS analysis on an Agilent 6530 accurate-mass quadrupole time-of-flight (Q-TOF) LC/MS (Agilent Technologies).

The sequences and modifications of oligonucleotides are shown in Supplementary Table S1.

### Analysis of oligonucleotides

The liquid chromatography analysis of siRNAs (using the sense strands) was performed on an Agilent 6530 accurate-mass Q-TOF using the following conditions: buffer A, 100 mM 1,1,1,3,3,3-hexafluoroisopropanol (HFIP) and 9 mM triethylamine (TEA) in LC-MS grade water; buffer B, 100 mM HFIP and 9 mM TEA in LC-MS-grade methanol; column, Agilent AdvanceBio oligonucleotides C18; 5–100% B 11 min; temperature, 60°C; flow rate, 0.5 ml/min. LC peaks were monitored at 260 nm.

### Cell culture

GBM8 primary human glioblastoma cells were received as frozen stocks from Dr. Miguel Sena-Esteves (UMass Chan Medical School), who obtained the original cells from Dr. Samuel Rabkin (Massachusetts General Hospital). GBM8 cells were previously transduced with lentivirus vector as described to generate GBM8 cells that constitutively express firefly luciferase (14). GBM6 primary human glioblastoma cells were received as frozen stocks from Dr. Anastasia Khvorova (UMass Chan Medical School); cells were originally collected from Timone Hospital (15). GBM8 and GBM6 cells were grown in suspension as tumorspheres in media consisting of an equal ratio of Neurobasal and D-MEM/F-12 media (Gibco) supplemented with working concentrations of 10× GlutaMAX-I, 100 mM HEPES Buffer solution, 10 mM sodium pyruvate, 1 mM MEM non-essential amino acids, 1× B27 supplement (Gibco), 2 μg/ml heparin (Sigma), 1× antibiotic-antimycotic solution (CellGro), 20 ng/ml recombinant human basic fibroblast growth factor (Peprotech), and 20 ng/ml recombinant human epidermal growth factor (Peprotech). Media for tumorsphere cultures was replaced with $\frac{1}{2}$ volume of fresh medium every other day by collecting and spinning down half the cell culture volume. Cells were passaged when the medium turned yellow-orange within 2 days of feeding. Cultures were maintained for 3–4 passages after thawing prior to all *in vitro* and *in vivo* experiments.

### Animal experiments

All animal procedures were conducted according to the Institutional Animal Care and Use Committee (IACUC) protocols of the University of Massachusetts Chan Medical School (IACUC protocol 202100196). Athymic nude female mice (6 weeks old, Jackson Laboratory, Bar Harbor Maine) were used in all tumor studies. FVB female mice (6–8 weeks old) were used to validate ASO or siRNA sequences (see Supplementary Figures S2 and S3). Mice were euthanized upon reaching experimental endpoint or humane endpoint, defined as the loss of >15% of initial body weight or exhibiting moribund symptoms (e.g. hunched posture, not able to right themselves, unresponsive to stimuli).

### Orthotopic tumor engraftment in mouse brain

Two days prior to GBM cell implantation, the medium of GBM tumorspheres was fully replaced with fresh medium to minimize clumping. On the day of implantation, GBM tumorspheres were resuspended in sterile Dulbecco's phosphate-buffered saline (1× DPBS, Gibco) and dissociated into single cells by gently triturating with a P1000 pipette. Animals were anesthetized and 50 000 cells in 1 μl were injected unilaterally into the left striatum (coordinates from bregma: +0.5 mm AP, +2.0 mm ML and −2.5 mm DV from skull surface) using stereotaxic frames. Cells were injected at a rate of 0.200 μl/min.

### *In vivo* GBM8 tumor xenograft imaging

For GBM8 and GBM6 cell lines used, initial natural progression studies were conducted to determine tumor growth kinetics. For GBM8 cells, tumor xenograft growth was measured weekly via *in vivo* bioluminescence imaging using IVIS Spectrum CT (Perkin-Elmer). Imaging data was acquired and quantified using Living Image 4.7.4 Software. One day prior to oligonucleotide injections, bioluminescence signals of GBM8 xenografts were quantified. Tumor-bearing mice were assigned to treatment groups based on tumor sizes such that each group contained an equivalent set of tumor sizes.

### Oligonucleotide delivery to orthotopic tumor xenografts

All oligonucleotides were resuspended in artificial cerebrospinal fluid (137 mM NaCl, 5 mM KCl, 2.3 mM CaCl_2_, 1.3 mM MgCl_2_, 20 mM glucose, 8 mM HEPES, pH 7.4 with NaOH) to the desired final concentration prior to *in vivo* administration. Tumor bearing mice received either unilateral or bilateral injections. For intracerebroventricular injections of ASO compounds, mice received a single injection into the lateral ventricle contralateral to the tumor (coordinates from bregma: −0.4 mm AP, −1.0 mm ML, −2.0 mm DV from skull surface). For intracerebroventricular injections of siRNA compounds, mice received bilateral injections into each lateral ventricle (coordinates from bregma: −0.4 mm AP, ±1.0 mm ML, −2.0 mm DV from skull surface). For injections into brain tissue, mice received bilateral injections of either ASO or siRNA compounds directly to the tumor or to the contralateral striatum (coordinates form bregma: +0.5 mm AP, ±2.0 mm ML and −2.5 mm DV from skull surface). Compounds were injected at a rate of 0.407 μl/second for intracerebroventricular injections or 0.200 μl/min for intratumoral/intrastriatal injections.

### Post-mortem tissue processing for oligonucleotide distribution and gene expression analysis

For brain biodistribution studies, tumor-bearing mice were injected with oligonucleotides as described above. After 48 h, mice were euthanized and perfused with 1× phosphate-buffered solution (Gibco) followed by 10% neutral buffered formalin. Brains were post-fixed in 10% neutral buffered formalin overnight at 4°C. Brains were paraffin-embedded and 4 μm sagittal or coronal sections were collected and stained with hematoxylin and eosin for pathology analysis or immmunostained (*see immunofluorescence section*).

For RNA silencing studies measured via quantitative real-time PCR (qPCR), mice were euthanized and brains were collected at the specified timepoint post-oligonucleotide injection. Brains were oriented in a stainless-steel brain matrix and 1 mm coronal sections were collected through the striatum. For every coronal section, a 2 mm biopsy punch was taken from the xenografted striatum, near the tumor implantation site, and the contralateral striatum. Tissue punches were stored in RNAlater overnight at 4°C. Individual punches were homogenized in Trizol using Fisherbrand Pellet Pestle Cordless Motor and RNase-free disposable pellet pestles (ThermoFisher Scientific). RNA was extracted using RNA Clean & Concentrator Kit (Zymo) and quantified on a Nanodrop (ThermoFisher Scientific).

For RNA silencing studies measured via QuantiGene (QG) 2.0 RNA Assay (Affymetrix), 1.5 mm tissue punches (three punches per tissue) were placed in QIAGEN Collection Microtubes holding 3 mm tungsten beads and lysed in 300 μl Homogenizing Buffer (Affymetrix) containing 0.2mg/ml Proteinase K (Invitrogen) using a QIAGEN TissueLyser II. Samples were then centrifuged at 1000 × g for 10 min and incubated for 1 h at 55–60°C.

### Measuring mRNA gene expression

For reverse transcription (RT), 1000 ng RNA was used with the High-Capacity cDNA Reverse Transcription Kit (ThermoFisher Scientific) according to the manufacturer's protocol.

For qPCR, individual reactions were performed using the following volumes: 2 μl of the RT reaction mixture (20 ng cDNA total), 10 μl of iTaq SuperMix (BioRad, Hercules, CA, USA), 1 μl 20× probe, and 7 μl of water for a final reaction volume of 20 μl. The qPCR reactions were conducted in technical duplicates on a CFX96 Real-Time System (BioRad) under the cycling conditions: initial denature at 95°C for 30 s, followed by 39 cycles of denaturation at 95°C for 5 s and extension at 60°C for 30 s.

All qPCR was done using primer-probe assays with 5′ FAM reporter and a 3′ ZEN/Iowa Black FQ quencher. Sequences were pre-designed and purchased from Integrated DNA Technologies (IDT, Coralville, Iowa, USA). Product assay IDs are follows: IDT pre-designed PrimeTime probes for human genes—*HTT* (Hs.PT.58.14833829), *APP* (Hs.PT.56a.27427814.g) and *HPRT (*Hs.PT.58v.45621572)—and mouse genes—*Htt* (Mm.PT.58.6953479), *App* (Mm.PT.58.11717878) and *Hprt* (Mm.PT.39a.22214828) (16).

For QG RNA analysis, tissue lysates and diluted probe sets (mouse App, mouse Ppib, or mouse Hprt) were added to the branched DNA (bDNA) capture plate, and signal was amplified and detected as described previously (17). Luminescence was detected on a Tecan M1000 (Tecan, Morrisville, NC).

### Immunofluorescence for brain sections

Paraffin-embedded sections were deparaffinized in two washes of xylene for 5 mins each. Sections were then rehydrated in a series of ethanol washes (100%, 95%, 70% and 50%) for 5 mins each, a brief wash in water, followed by two washes in 1× TBST. For ASO antibody staining only, sections were treated with Dako Proteinase K for 10 min at room temperature prior to the next step. Sections were treated with an application of Cyto-Q Background Buster (Innovex Biosciences) for 30 min at room temperature (RT). After washing twice in 1X TBST, primary antibody (*see table below*) diluted in antisera (2% BSA, 10% normal goat serum, 0.025% Triton X-100 in 1× TBST) was applied. The slides were incubated with diluted antisera overnight at 4°C. The next day, slides were washed 3 times for 5 min each with 1× TBST. Secondary antibodies were applied to slides (diluted 1:5000, Cell Signaling Technology) and incubated 1 h at RT protected from light. Following secondary antibody incubation, slides were washed 3 times for 5 min each with 1× TBST. Hoescht dye was used for nuclear counterstaining; Prolong Gold Antifade (ThermoFisher P36930) was applied as mountant prior to cover slipping. Microscopy images were acquired using the Leica DMi8 inverted microscope. Consistent imaging parameters were maintained across all slides.

**Table:** 

Primary antibody (Ab)	Catalog #, manufacturer	Dilution factor
Anti-human Ab	MAB4383, EMD Millipore	1:150
PS-ASO Ab	Developed in house	1:1000

### Graphs and statistical analyses

Data were analyzed using GraphPad Prism 10.1.2 software for Windows (GraphPad Software, Inc., San Diego, CA). For each independent mouse experiment, the levels of human or mouse mRNA silencing from a specific brain region in treated groups were normalized to the mean of the human RNA or mouse RNA from the corresponding region in the vehicle control groups respectively (or NTC group if a vehicle group was not included). In vivo data were analyzed using a one-way ANOVA with a post hoc Tukey multiple comparisons test. Asterisks (*) denote human gene expression significance relative to human NTC or vehicle control (*P< 0.05, **P< 0.01, ***P< 0.001, ^****^P< 0.0001), hash symbols (#) denote mouse gene expression significance relative to mouse NTC or vehicle control (^#^*P*< 0.05, ^##^*P*< 0.01, ^###^*P*< 0.001, ^####^*P*< 0.0001), and ns = not significant. Graphs are plotted as bar graphs (mean ± standard deviation).

## Results

### Measuring oligonucleotide activity in patient-derived orthotopic GBM tumor xenograft models

**Figure 1.**
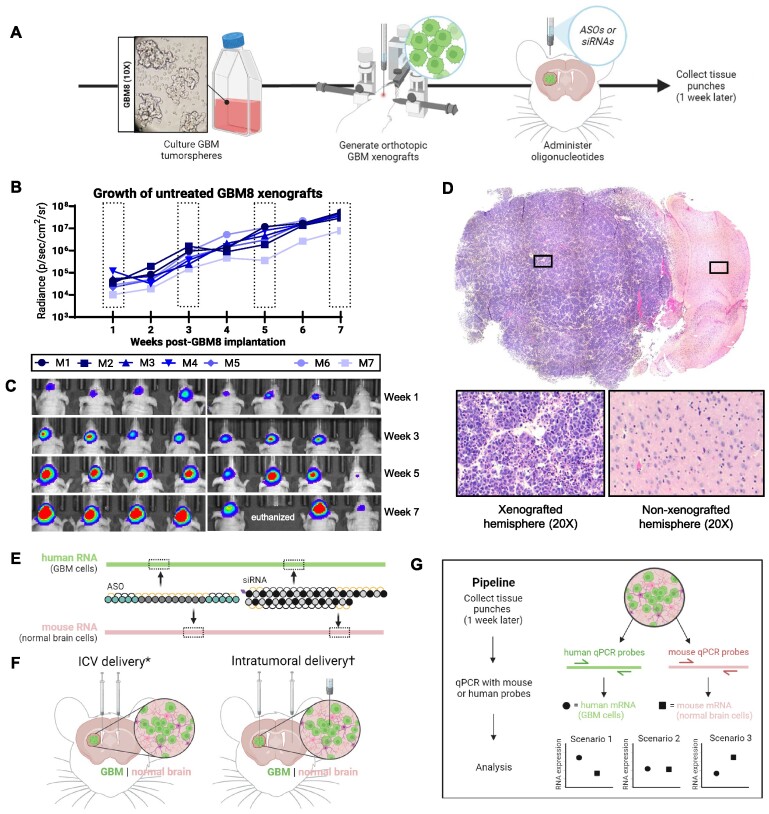
Differentiating oligonucleotide-induced gene silencing using orthotopic, patient-derived GBM xenograft models. (**A**) Schematic of pipeline used to generate orthotopic GBM8 xenografts. (**B**) Weekly measurements of bioluminescence from untreated GBM8 xenografts 1 week after GBM8 cell implantation to humane endpoint, dotted boxes indicate highlighted timepoints for (**C**) corresponding bioluminescence imaging of tumor burden at 1, 3, 5 and 7 weeks post-GBM8 implantation. (**D**) Representative 10× tile scan images of hematoxylin-and-eosin staining of GBM8 xenograft coronal sections at humane endpoint; insets show highlighted regions in xenografted and non-xenografted hemispheres. (**E**) Cross-reactive ASOs or siRNAs bind homologous sequences within homologous human (GBM cells) and mouse (normal brain cells) RNA. (**F**) Oligonucleotides were delivered via intracerebroventricular injections or intratumoral injections; (*) unilateral ICV injections were used to deliver ASOs while bilateral ICV injections were used to deliver siRNAs. (^†^) bilateral injections were used to deliver ASOs or siRNAs directly to the tumor and the contralateral striatum. (**G**) Pipeline depicting tissue sample collection, processing, and analysis 1-week post-oligonucleotide injections. Created with BioRender.com.

To study oligonucleotide-induced silencing in brain tumors, we developed a custom real-time quantitative PCR (qPCR) assay using a patient-derived, orthotopic GBM xenograft model. Here, patient-derived GBM cells are directly implanted into the striatum in the brains of immunocompromised mice (Figure 1A). The primary cell line we used for *in vivo* studies is called GBM8, a stem cell-like cell line grown in suspension *in vitro* (Supplementary Figure S1) that readily generates highly invasive GBM tumor xenografts *in vivo* (14,18–20). This line was previously transduced with a lentivirus vector to constitutively express firefly luciferase to enable real-time measurements of tumor size (14). Following optimized parameters, GBM8 xenografts grow over an 8-week period after initial implantation (Figure 1B and C). Most mice require humane euthanasia before reaching the end of week 8. Therefore, the corresponding collection of tumor burden images (Figure 1C) represents the most complete set of images prior to humane euthanasia which occurred throughout week 8. These natural progression studies of untreated tumors (Figure 1B and C) indicate that the tumors grow steadily in the first 2 weeks, followed by an exponential-growth phase in week 3–4 post-cell implantation. By 4 weeks post-cell implantation, tumors resume steady and consistent proliferation until the mouse reaches humane endpoint at approximately 8 weeks post-cell implantation. Post-mortem tissue analysis of GBM8 xenografts at humane endpoint show pathological features reminiscent of patient GBM tumors, including extensive tissue infiltration, hyper-nucleation, regions of pseudopalisading cells and necrotic foci (Figure 1D) (21).

Determining activity of therapeutic oligonucleotides in the target tissue or cell population is key to understanding therapeutic mechanism of action and moving the drug towards the clinic. For example, in complex tissues such as lung (22) and brain (23), oligonucleotides have been observed to show strikingly different activity in different cell populations. We reasoned that patient-derived tumor xenografts provide a straightforward approach to quantify oligonucleotide-induced silencing in tumors *in vivo* because of species-based sequence differences between the donor and host cells. Based on this principle, we developed an approach to distinguish gene silencing in tumor cells versus normal cells using human- or mouse-specific qPCR probes respectively (Figure 1E and G).

In particular, we considered that we could deliver an oligonucleotide that targets a conserved region of a homologous gene to silence the RNA in both mouse and human cells (Figure 1E). We selected *huntingtin* as our primary gene target. *Huntingtin* is expressed by our patient-derived GBM cell lines as well as ubiquitously in mouse brain by the major CNS cell types (i.e. neurons, astrocytes, and oligodendrocytes). Furthermore, silencing *huntingtin* should not affect the viability of either GBM cells or normal brain cells, an outcome that would introduce bias in distinguishing oligonucleotide activity. Hereafter, human *huntingtin* (in GBM xenograft cells) will be identified as ‘*HTT*’, and normal mouse brain *huntingtin* will be identified as ‘*Htt’*. To confirm results were not *HTT/Htt* specific, we chose *amyloid-beta precursor protein* as a second target using the same target selection criteria as *huntingtin*. Similarly, human GBM xenograft *amyloid-beta precursor protein* will be identified as ‘*APP*’ and normal mouse brain cells as ‘*App*’. The same treatment timepoints and treatment duration previously described for *HTT/Htt* in the GBM8 model were applied to *APP/App* experiments as well. We used previously validated ASO or siRNA sequences against homologous sequences in *HTT/Htt* (12,24) and designed a new siRNA sequence targeting homologous regions of *APP/App* (25). We confirmed that ASOs and siRNAs were active in silencing both the human and mouse isoforms of their target genes in cell culture models (Supplementary Figures S2 and S3).

To confirm that our qPCR was able to independently quantify human and mouse transcripts from an infiltrative xenograft model (mixed cell population), we mixed human cell-derived and mouse cell-derived RNA at different ratios prior to reverse transcription. Gene expression was measured using qPCR probes that were validated to be specific to either species. We observed that the qPCR quantification from this series of mixed samples correlated well with the RNA input ratios (Supplementary Table S2). This justified our approach to specifically measure silencing in a mixed population of tumor cells and normal brain cells as is found in an infiltrative xenograft model (Figure 1D, Supplementary Figure S1).

For all xenograft experiments, we selected a short treatment timepoint (harvesting tissue punches 1 week after ASO/siRNA administration, unless otherwise indicated) to minimize the risk that our results would be confounded by dilution of drug activity caused by tumor proliferation. As shown in Figure 1F, we injected ASOs or siRNAs intracranially via one of two administration routes (*see below*) when tumor xenografts consisted of both GBM cells and normal brain cells and were approximately half-way to humane endpoint, based on the kinetics data shown in Figure 1B and C.

### MOE-modified ASO gapmers show poor gene silencing in GBM cells compared to normal brain cells

**Figure 2.**
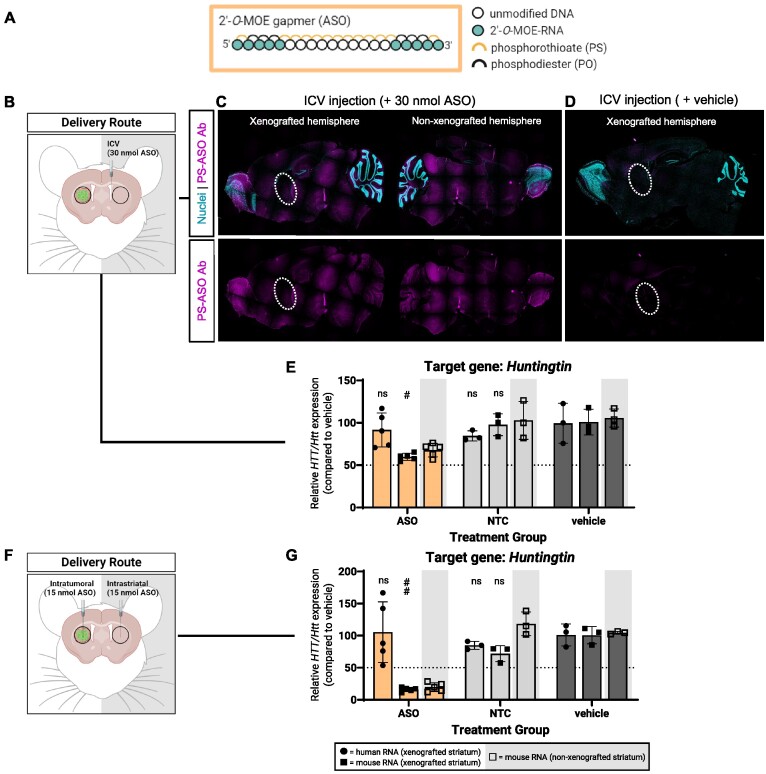
Partial PS, MOE-modified ASO gapmers show less silencing activity in GBM cells over normal brain cells, independent of delivery route. (**A**) Chemical pattern and position of modifications featured in the ASO gapmers used in this study. (**B**) For unilateral ICV injections of ASO gapmers: (**C**) representative sagittal sections of xenografted (xenograft location denoted by dashed circle) and non-xenografted hemispheres of ASO injected mouse, (**D**) sagittal section of xenografted hemisphere of vehicle injected mouse, and (**E**) silencing of human *HTT* and mouse *Htt* mRNA from xenografted and non-xenografted striata measured using qPCR. (**F**) For bilateral intratumoral and intrastriatal injections of ASO gapmers: (**G**) silencing of human *HTT* and mouse *Htt* mRNA from xenografted and non-xenografted striata measured using qPCR. NTC = non-targeting ASO control. Created with BioRender.com.

We first applied our assay to determine the activity profile of a 2′-*O*-MOE, partially PS-modified ASO gapmer, a chemical design that is routinely applied to ASOs in clinical development for CNS diseases (Figure 2A). We delivered on-target ASOs (ASO*^HTT/Htt^*), non-target ASO control (NTC) which is not expected to impact gene expression), or vehicle control (artificial cerebrospinal fluid) to xenografts using intracerebroventricular or intratumoral injections.

For intracerebroventricular (ICV) injections, the oligonucleotide is injected into endogenous cerebrospinal fluid (CSF) via the lateral ventricles. A single, unilateral ICV injection is sufficient to deliver ASOs to both brain hemispheres. A single, unilateral 30 nmol ASO dose was administered to the right lateral ventricle (Figure 2B).

We collected samples 1 week later to assess ICV efficiency using two methods. In our first approach, we assessed ASO tissue distribution by staining sagittal brain sections with an antibody against PS linkages in the modified ASOs. We detected uniform ASO distribution in both the xenografted and non-xenografted hemispheres (Figure 2C) suggesting that the tumor had not impacted ASO delivery. No ASO signal was detected in vehicle injected, xenografted hemispheres (Figure 2D). In our second approach, we took tissue punches from the xenografted and non-xenografted striata and measured *huntingtin* RNA levels. We observed a 10% *HTT* reduction in human mRNA and a 40% *Htt* reduction in mouse mRNA (Figure 2E). Given that GBM8 cells and normal mouse brain cells originated from the same tissue punches, we concluded that the difference in target reduction was not related to differences in ASO exposure.

Efficient ICV injections require unobstructed CSF flow; a rapidly growing brain tumor could distort the lateral ventricles and other CSF pathways which could inhibit oligonucleotide delivery. To confirm that the silencing was not overly influenced by tumor-induced changes in CSF dynamics, we measured *Htt* levels from normal brain cells in the non-xenografted hemisphere. Here, we also observed a 40% silencing of mouse *Htt* mRNA, equivalent to the silencing in normal brain cells from the xenografted striatum (Figure 2E), functionally confirming that ICV administration of ASOs is not impaired by tumor xenografts at this point in the tumor timeline.

We also delivered ASO*^HTT/Htt^* by intratumoral injection as a second delivery method to further ensure that our results were not related to CSF flow or injection-related artifacts. Unlike with ICV delivery, oligonucleotides delivered intratumorally are retained locally in the tissue around the site of injection and do not distribute to the contralateral hemisphere. Therefore, for internal comparison, mice receiving intratumoral injections received a second injection of an equivalent ASO dose delivered to the contralateral, non-xenografted striatum (Figure 2F).

In mice intratumorally injected with ASOs*^HTT/Htt^*, we did not measure significant reductions in human *HTT* mRNA; in contrast, we measured an 85% reduction in mouse *Htt* mRNA (Figure 2G). This reduction in *Htt* was approximately double that of what was observed in mice ICV injected with ASOs*^HTT/Htt^* (Figure 2E). Intratumoral injections result in greater local ASO concentrations around the injection site; greater silencing can be achieved despite the lower ASO dose used. Thus, while the observed dramatic increase in reduction in mouse *Htt* mRNA following intratumoral ASOs*^HTT/Htt^* injections was expected, the fact that it was exclusive to mouse *Htt* (and not human *HTT*) was not anticipated. These results suggest there may be a difference in functional ASO uptake comparing normal brain cells and GBM cells. Levels of mouse *Htt* mRNA showed comparable reductions in both the xenografted and non-xenografted striata (Figure 2G).

**Figure 3.**
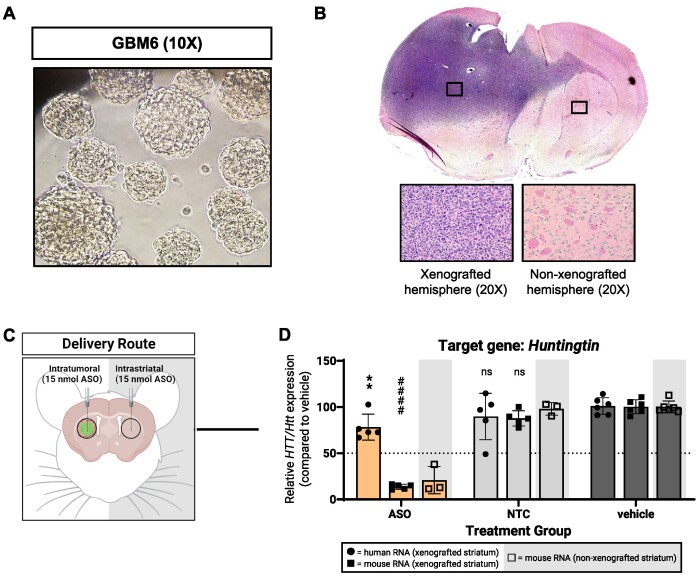
Partial PS, MOE-modified ASO gapmers show less silencing activity in GBM cells over normal brain cells in a second patient-derived xenograft model (GBM6). (**A**) Human GBM6 tumorspheres in culture 3 days after thawing from cryopreservation. (**B**) Representative 10X tile scan image of hematoxylin-and-eosin stained coronal section from GBM6 xenograft at humane endpoint; insets show highlighted regions of xenografted and non-xenografted hemispheres. (**C**) For bilateral injections of ASO gapmers: (**D**) silencing of human *HTT* and mouse *Htt* mRNA from xenografted and non-xenografted striata measured using qPCR. Created with BioRender.com.

GBM8 cells were derived from a patient tumor classified as proneural subtyped GBM (26,27). To ensure the observed differential silencing trends were neither cell line nor tumor subtype specific, we tested ASO*^HTT/Htt^* in a GBM6 xenograft model, where GBM6 cells (Figure 3A) were derived from a patient with a mesenchymal-like subtyped GBM (15,28,29). GBM6 xenografts recapitulate clinically relevant GBM tumor features *in vivo* but their progression is distinct from GBM8 xenografts based on natural tumor progression studies and post-mortem xenograft pathology (Figure 3B). GBM6 cells do not express luciferase so tumor xenografts could not be size-matched prior to treatment.

We injected 15 nmol of ASO*^HTT/Htt^* or vehicle volume equivalent directly into established GBM6 xenografts (Figure 3C). In GBM6 xenografts, we observed a 22% reduction in human *HTT* mRNA and an 85% reduction in mouse *Htt* mRNA (Figure 3D), recapitulating the silencing patterns we initially observed in GBM8 xenografts. These results indicate that the weaker ASO silencing is not intrinsically limited to GBM8 or proneural xenografts and that the ASOs may not be chemically optimized for strong activity in GBM cells.

### Unconjugated, asymmetric monovalent and divalent siRNAs show less silencing in GBM cells compared to normal brain cells

**Figure 4.**
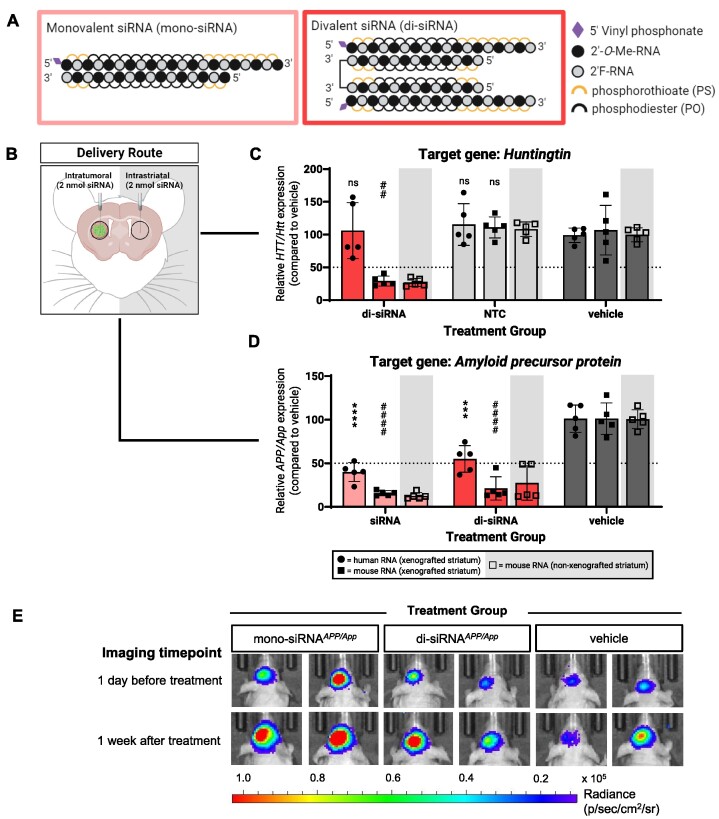
Unconjugated, partial PS-modified siRNAs show less silencing activity in GBM cells compared to normal brain cells. (**A**) Chemical pattern and position of modifications featured in the unconjugated siRNAs used in this study. (**B**) For bilateral intratumoral and intrastriatal injections of unconjugated siRNAs: (**C**) silencing of human *HTT* and mouse *Htt* mRNA or (**D**) silencing of human *APP* and mouse *App* mRNA from xenografted and non-xenografted striata measured using qPCR. (**E**) Representative bioluminescence images of GBM8 xenograft burden 1 day before treatment and 1 week after treatment; two mice shown per condition. NTC = non-targeting siRNA control. Created with BioRender.com.

Small-interfering RNAs (siRNAs) induce gene silencing through the RNAi pathway, a mechanism distinct from that of ASOs. While monovalent siRNAs (mono-siRNA) have demonstrated efficacy in the brain, next-generation divalent siRNAs (di-siRNA) scaffolds show improved pharmacology over their monovalent counterparts and are thus one of the lead siRNA platforms in development for use in the CNS (12). The di-siRNAs used in these studies consist of two chemically modified, asymmetric mono-siRNAs of the same sequence and chemistry connected by a linker via the 3′ end of the sense strands (in experiments comparing mono-siRNAs to di-siRNAs, dose was determined using the concentration of the antisense strand) (Figure 4A). Considering their mechanistic and chemical differences from ASOs, we wanted to test whether siRNAs would be a more efficient way to silence genes in GBM cells.

We used a previously validated di-siRNA that efficiently targets both human *HTT* mRNA and mouse *Htt* mRNA transcripts (di-siRNA*^HTT/Htt^*) (12). This di-siRNA binds its target RNA in a different region than the tested ASO*^HTT/Htt^* (Figure 1E, Supplementary Table S1). Di-siRNAs were delivered intratumorally at a dose of 2 nmol to GBM8 xenografts (Figure 4B). After 1 week, the di-siRNA induced relatively minimal reduction of human *HTT* mRNA, but a strong reduction of mouse *Htt* mRNA (∼70% *Htt* silencing, Figure 4C). Levels of mouse *Htt* mRNA showed comparable reductions in both the xenografted and non-xenografted striata (Figure 4C).

This greater silencing in normal brain cells than in GBM cells by siRNAs remains consistent with the silencing patterns we observed with ASO*^HTT/Htt^*. Obtaining consistent results across two RNA silencing modalities targeting different homologous *HTT/Htt* sequences suggests that the weaker silencing in GBM cells is likely not related to the oligonucleotide sequence or effector (RNase H or Ago) activity. While ASOs are a stronger choice for silencing nuclear transcripts and siRNAs for silencing cytoplasmic transcripts, observing the same trend for both oligonucleotide modalities also suggests that the silencing trends are not due to target mRNA localization.

Target-intrinsic factors such as post-transcriptional processing and secondary structure have been shown to affect efficacy of antisense therapeutics (30–32). To assess how generalizable these results were, we tested unconjugated, asymmetric monovalent siRNA (mono-siRNA) and di-siRNA compounds (Figure 4A) while targeting a second gene: *amyloid-beta precursor protein* (human *APP/* mouse *App*) (25).

We injected 2 nmol of each siRNA compound intratumorally; the contralateral striatum also received an equivalent dose of the respective siRNA (Figure 4B). After 1 week, we observed that mono-siRNA*^APP/App^* resulted in 60% reduction in human *APP* mRNA and an 85% reduction in mouse *App* mRNA (Figure 4D). Di-siRNA*^APP/App^* resulted in 45% reduction in human *APP* mRNA and 80% in mouse *App* mRNA (Figure 4D). The higher silencing observed in mouse cells relative to tumor cells is consistent with the results we obtained with the *HTT/Htt* series, but the difference in silencing was less pronounced. Levels of mouse *App* mRNA showed comparable reductions in both the xenografted and non-xenografted striata (Figure 4D).

Due to its role in inflammation and cell proliferation, *amyloid-beta precursor protein* has been implicated in several malignant cancers including GBM (33–35). By measuring GBM8 xenograft sizes prior to and after siRNA treatment, we confirmed that *APP* silencing did not induce changes in xenograft growth relative to the vehicle treated group (Figure 4E).

Results from the ASO and siRNAs we tested suggest that traditional chemistries optimized for functional activity in normal brain show less functional activity in GBM cells compared to normal brain cells. Having developed this assay to quantitate silencing in GBM versus normal brain cells, we next set out to apply this assay to identify oligonucleotide chemistries that are more consistently active in the context of GBM tumors.

### Panel of lipid-conjugated siRNAs induce gene silencing in brain tumors but show differential toxicity profiles in CNS

**Figure 5.**
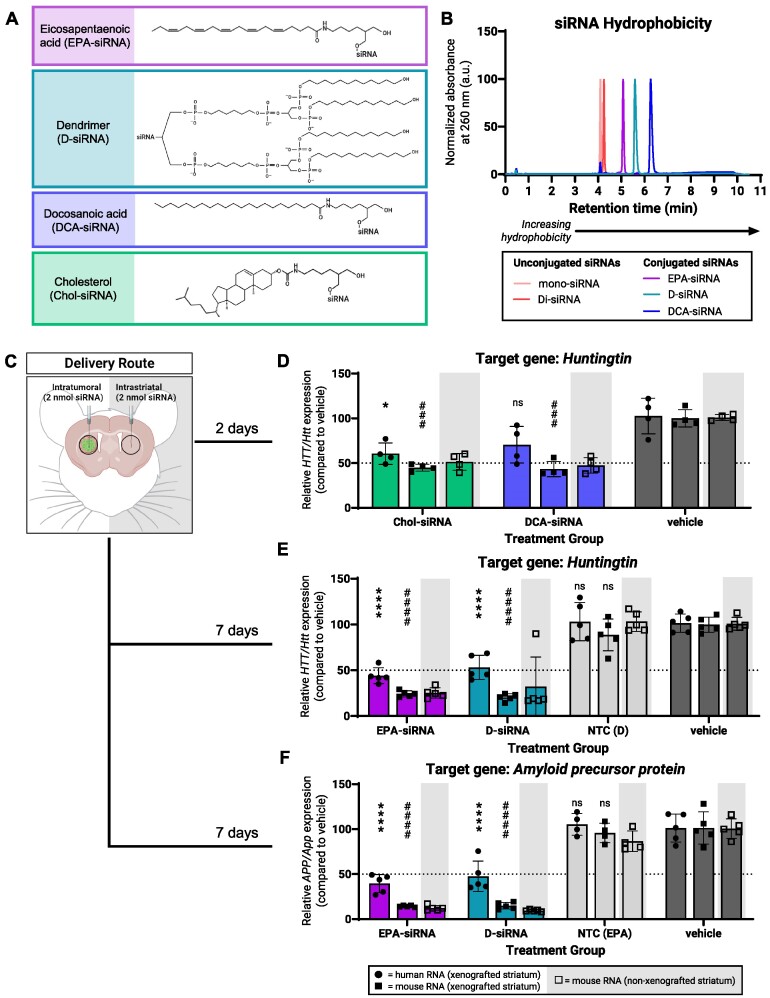
Lipid-conjugated siRNAs show modest silencing in GBM xenografts that is consistent across two mRNA targets. (**A**) Structural formulas of lipids conjugated to siRNAs. (**B**) Liquid chromatography spectra of selected unconjugated and conjugated siRNA*^HTT/Htt^* sense strands showing differences in retention time (hydrophobicity). (**C**) For bilateral intratumoral and intrastriatal injections of lipid-conjugated siRNAs: (**D**) silencing of human *HTT* and mouse *Htt* mRNA with Chol-siRNA or DCA-siRNA, (**E**) silencing of human *HTT* and mouse *Htt* mRNA with EPA-siRNA or D-siRNA, and (**F**) silencing of human *APP* and mouse *App* mRNA with EPA-siRNA and D-siRNA from xenografted and non-xenografted striata measured using qPCR. Created with BioRender.com.

Ligand conjugation—attaching lipids, sugars, peptides, aptamers, or other targeting molecules to an oligonucleotide—is one strategy used to enhance delivery (36–38). Lipids are a specific class of conjugates that can enhance oligonucleotide activity by prolonging tissue retention, promoting cell uptake, and enhancing endosomal escape (10,11,39–43). siRNAs modified with cholesterol ligands showed efficient gene silencing in GBM xenografts (19). We decided to explore a broader panel of lipid conjugates to improve functional delivery to GBM cells; we tested four lipid-conjugated siRNAs that have been previously characterized in CNS or systemic, extra-hepatic tissues (Figure 5A) (11,19,43–47). Three of the four conjugates were biologically relevant lipids: cholesterol (Chol-siRNA), docosanoic acid (DCA-siRNA), a 22-carbon chain saturated fatty acid, and eicosapentaenoic acid (EPA-siRNA), a 20-carbon chain unsaturated fatty acid. The fourth conjugate is a synthetic, dendrimeric structure (D-siRNA) that has shown high binding to albumin (13,46–48). Each conjugate was covalently attached to an asymmetric, chemically-modified monovalent siRNA scaffold via the 3′ (Chol, DCA and EPA) or 5′ (dendrimer) end of the sense strand.

Chemical composition of the lipid conjugate impacts oligonucleotide hydrophobicity which can influence subsequent tissue accumulation and functionality (11,44). To assess hydrophobicity, we used liquid chromatography (LC) to analyze the retention time of selected siRNAs (Figure 5B). As expected, unconjugated siRNAs had the shortest retention times indicating they were the least hydrophobic siRNAs in the panel. EPA-, D- and DCA-siRNA showed longer retention times demonstrating greater hydrophobicity relative to the unconjugated siRNAs. While Chol-siRNA was not directly analyzed here, we expect this siRNA to have the longest retention time (highest hydrophobicity) based on previous results which included a similar panel of lipid-conjugated siRNAs (11).

For *in vivo* studies, mice were intratumorally injected with 2 nmol of lipid-conjugated siRNA (Figure 5C). Mice injected with Chol-siRNA*^HTT/Htt^* and DCA-siRNA*^HTT/Htt^* exhibited phenotypic hallmarks of acute oligonucleotide-induced neurotoxicity (49–51). These phenotypes included seizures, hyperactivity and ataxia which started immediately following anesthetic reversal and were more severe and durable than the reversible motor phenotypes seen after injection of some oligonucleotides (Moazami *et al. bioRxiv* 2021). Unfortunately, the condition of these mice did not improve, and the mice were humanely euthanized before reaching the planned experimental timepoint. Consequently, mRNA levels from these mice were measured 2 days after injections rather than 1 week.

Chol-siRNA*^HTT/Htt^* and DCA-siRNA*^HTT/Htt^* reduced human *HTT* mRNA (i.e. in tumor xenograft) by 35% and 30%, respectively (Figure 5D). Both compounds still showed greater silencing in normal brain cells reducing mouse *Htt mRNA* in the xenograft by approximately 55% (Figure 5D). Levels of mouse *Htt* mRNA showed comparable reductions in both the xenografted and non-xenografted striata (Figure 5D). The extent of *HTT/Htt* mRNA reduction observed after 2 days likely does not reflect the full extent of silencing that could be achieved in either GBM or normal brain cells using these siRNA compounds since maximal silencing *in vivo* normally requires longer treatment. While we were encouraged that Chol- and DCA-siRNA did improve *HTT* silencing in GBM cells compared to ASOs*^HTT/Htt^* and unconjugated mono- and di-siRNA*^HTT/Htt^*, their neurotoxicity severely limits their therapeutic index. As a result, we did not test Chol-siRNA or DCA-siRNA against *APP/App* and do not recommend their use in brain tumor studies (*see discussion*).

By contrast, mice intratumorally injected with the less hydrophobic conjugates EPA-siRNA*^HTT/Htt^* or D-siRNA*^HTT/Htt^* did not show major signs of oligonucleotide-induced neurotoxicity (*see discussion*). mRNA levels from these mice were measured after 1 week. EPA-siRNA*^HTT/Htt^* reduced human *HTT* mRNA by 56% while D-siRNA*^HTT/Htt^* efficiently reduced human *HTT* mRNA by 47% (Figure 5E). As such, these two moderately hydrophobic conjugates provided the strongest silencing observed so far against *HTT* transcripts (i.e. in tumor xenografts). EPA- and D-siRNA silencing remained greater in normal brain cells, reducing mouse *Htt* mRNA levels by 80% and 75% respectively (Figure 5E). Levels of mouse *Htt* mRNA again showed comparable reductions in both the xenografted and non-xenografted striata (Figure 5E).

To explore whether this was applicable to other siRNA modification patterns, we tested an additional chemical pattern (termed ‘pattern 2’) conjugated to the amphiphilic dendrimer (Supplementary Figure S4A). We injected these compounds featuring chemical modification pattern 2 intratumorally at 2 nmol (Supplementary Figure S4B). Compared to the initial chemical modification pattern used for the siRNAs featured in main text figures, pattern 2 does not have uniformly alternating 2′-*O*-Me-RNA/2′F-RNA modifications. We found that while pattern 2 was overall less efficacious in comparison to the initial modification pattern (33% *HTT* mRNA silencing in tumor, 70% *Htt* mRNA silencing in normal brain cells), pattern 2 still showed greater mRNA silencing in normal brain cells than in tumor cells (Supplementary Figure S4C).

To test whether this effective silencing would also be applicable to other targets, as above, we then tested EPA-siRNA and D-siRNA against our second target, *APP/App*. After 1 week, *APP* levels in human tumor cells were reduced by >50% for both EPA-siRNA*^APP/App^* and D-siRNA*^APP/App^* (Figure 5F). Levels of mouse *App* mRNA showed comparable reductions in both the xenografted and non-xenografted striata (Figure 5F). These results demonstrate that EPA and amphiphilic dendrimer conjugates provide levels of gene silencing in GBM xenograft cells *in vivo* that are comparable or stronger than unconjugated ASOs and siRNAs (Figures 2G, 3D and 4C, D).

### Amphiphilic and moderately hydrophobic lipid-conjugated siRNAs can be functionally delivered to xenografts using different CNS delivery routes

Intratumoral delivery is a surgically feasible method to administer drugs to GBM tumors. If operable, however, the bulk tumor mass is usually resected thereby reducing the clinical utility of these injections. Infiltrative GBM cells—tumor cells that have migrated away from the tumor core into adjacent normal brain tissue—are nearly impossible to resect and ultimately drive tumor recurrence (52). A delivery method that facilitates broad oligonucleotide distribution through brain tissue to reach refractory, migrating GBM cells is therefore necessary to achieve clinical success in treating GBM.

ICV injections in mice and intrathecal injections in patients can deliver oligonucleotides to the brain and spinal cord. Unconjugated, partial PS-modified ASOs and siRNAs are hydrophilic which helps promote distribution in CSF. With lipid conjugates, EPA-siRNA and D-siRNA are more hydrophobic and it was not known how this increased hydrophobicity would affect CSF-mediated distribution. To test this, we administered selected *huntingtin* or *amyloid precursor protein* targeting siRNAs to GBM8 xenografts via bilateral, bolus ICV injections and measured gene expression 1 week later.

**Figure 6.**
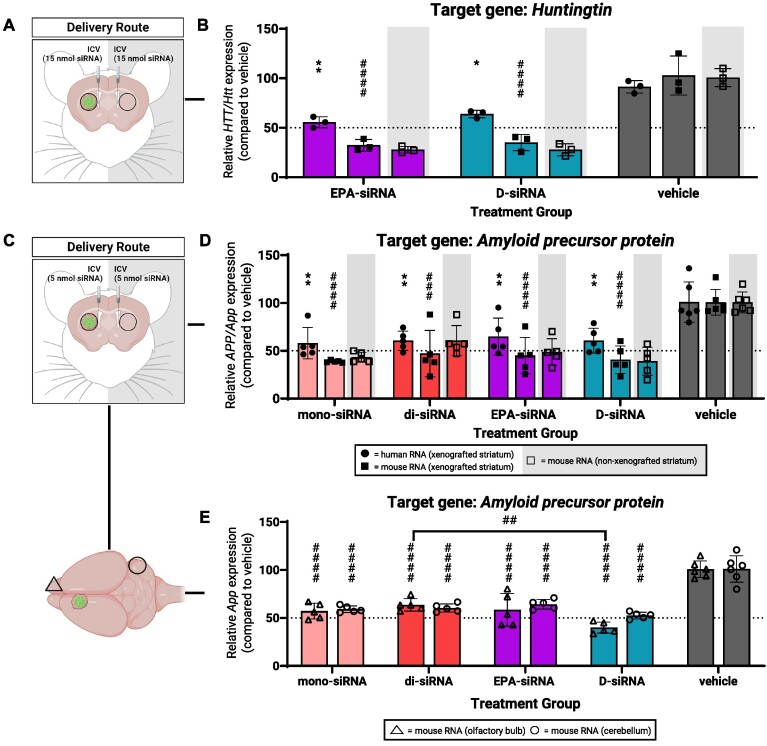
Lipid-conjugated siRNAs can be functionally delivered to GBM8 xenografts via ICV injections. (**A**) For bilateral ICV injections of 30 nmol total siRNA: (**B**) silencing of human *HTT* and mouse *Htt* mRNA from xenografted and non-xenografted striata measured using qPCR. (**C**) For bilateral ICV injections of 10 nmol total siRNA silencing of human *APP* and mouse *App* mRNA from (**D**) xenografted and non-xenografted striata and (**E**) olfactory bulb and cerebellum measured using qPCR. Created with BioRender.com.

For *huntingtin* ICV studies, EPA-siRNA*^HTT/Htt^*or D-siRNA*^HTT/Htt^*were injected at a total dose of 30 nmol (Figure 6A). EPA-siRNA*^HTT/Htt^*reduced human *HTT* mRNA by 45% and corresponding mouse *Htt* mRNA by 68% (Figure 6B). D-siRNA*^HTT/Htt^* reduced human *HTT* mRNA levels by 36% and mouse *Htt* mRNA levels by 65% (Figure 6B). The extent of human *HTT* and mouse *Htt* mRNA reduction following ICV delivery was ∼10% lower relative to the extent of reduction observed following intratumoral delivery (Figure 5E). This difference is not surprising and is likely a function of lower local drug concentrations compounded by the xenograft being located in the striatum, a deep brain region that is less accessible to oligonucleotides via CSF flow, even in healthy brains.

For *amyloid precursor protein* ICV studies, siRNAs were injected at a total dose of 10 nmol (Figure 6C). We used a lower dose than was used for the *Huntingtin* ICV studies to ensure that we were not using a saturating dose. We also included asymmetric, unconjugated mono- and di-siRNA in this experiment since these compounds are expected to distribute broadly in brain tissue and can therefore be used as standards to compare distribution efficiency of lipid conjugated siRNAs. After 1 week, the tested siRNAs reduced human *APP* mRNA levels by more than 35% and mouse *App* mRNA levels by more than 50% (Figure 6D).

To ensure efficient ICV administration, we measured mouse mRNA levels in the contralateral, non-xenografted striata as done previously for ASO*^HTT/Htt^*(Figure 2E). Levels of mouse *Htt* and *App* mRNA showed comparable reductions in both the xenografted and non-xenografted striata (Figure 6B and D).

To assess how broadly EPA-siRNA and D-siRNA distributed in the brain, we measured mouse *App* expression in the olfactory bulb and the posterior lobe of the cerebellum, two of the furthest regions along the anterior-posterior axis of the mouse brain. EPA-siRNA*^APP/App^* reduced mouse *App* mRNA levels by 40% (olfactory bulb) and 35% (cerebellum) with mono- and di-siRNA*^APP/App^* showing similar reductions (Figure 6E). Encouragingly, the amphiphilic D-siRNA*^APP/App^*also showed functional silencing, reducing mouse *App* mRNA levels by 45% in the cerebellum. In the olfactory bulb in particular, D-siRNAs reduced mouse *App* mRNA levels by 60% which slightly better than unconjugated, asymmetric di-siRNAs (Figure 6E).

Overall, these findings demonstrate that slightly hydrophobic EPA-siRNAs or amphiphilic D-siRNAs can be functionally delivered to GBM xenografts via a delivery route that supports drug distribution to distal tumor cells driving recurrent GBM. Furthermore, the functional distribution of EPA- or D-siRNAs is comparable to that of unconjugated, asymmetric siRNAs suggesting that conjugation of these particular lipids does not inhibit efficient distribution in brain tissue. Broad functional distribution, together with consistent gene silencing across two different mRNA targets in GBM cells, warrant further investigation into the potential applications of EPA- and D-siRNA as GBM therapeutics.

### Folate conjugated ASOs do not dramatically improve gene silencing in GBM8 xenografts

**Figure 7.**
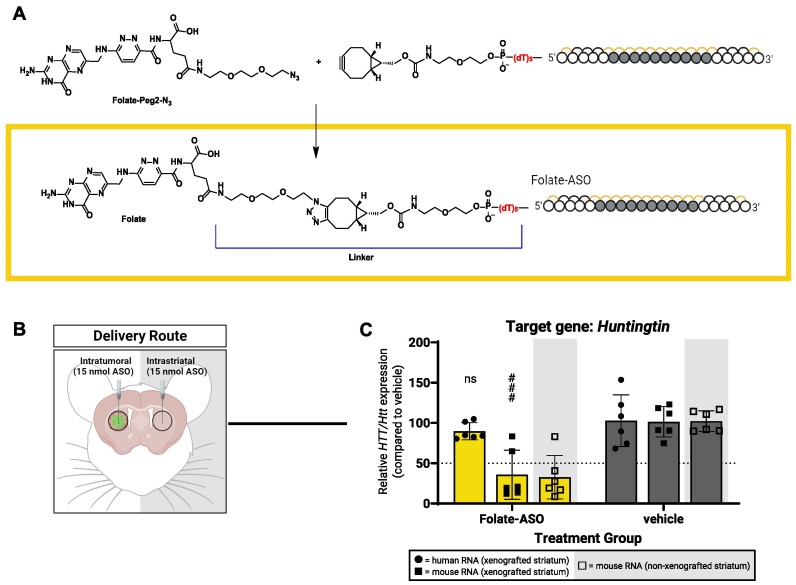
Folate conjugated ASO gapmers show less silencing activity in GBM cells compared to normal brain cells in GBM8 xenografts. (**A**) Synthesis scheme for folate conjugated ASOs. (**B**) For bilateral injections of folate-ASOs: (**C**) silencing of human *HTT* and mouse *Htt* mRNA measured using qPCR. Created with BioRender.com.

Several studies have used targeted delivery approaches by attaching a ligand—whose corresponding receptor is overexpressed on cancer cells—to the oligonucleotide to promote tumor uptake (53–60). To test this, we synthesized a folate conjugated ASO*^HTT/Htt^* (Folate- ASO*^HTT/Htt^*) (Figure 7A) based on the rationale that certain GBMs overexpress folate receptors and transporters to meet the *de novo* DNA synthesis demands of a rapidly proliferating tumor (61,62). However, Folate-ASO*^HTT/Htt^* did not improve silencing in GBM cells compared to unconjugated ASO*^HTT/Htt^*following intratumoral delivery (Figure 7B and C). Selective cell delivery of folate conjugates typically requires ∼10^5^–10^6^ copies of high-affinity folate receptors per cell, which we suspect was not the case for our specific GBM model. We measured the expression of *folate receptor alpha* (*FRα*) (63) specifically in GBM8 xenografts but did not observe higher *FRα* expression compared to normal brain tissue. We recognize however, that cells have several ways to take up folate and *FRα* represents just one of these ways (64). Thus, while targeted approaches are an optimal choice for drug delivery, this strategy may be more challenging for tumors with high intratumoral heterogeneity which could then later develop resistance to the drug by reducing expression of the specific receptor.

### Tumor proliferation does not drive differences in oligonucleotide-mediated gene silencing

**Figure 8.**
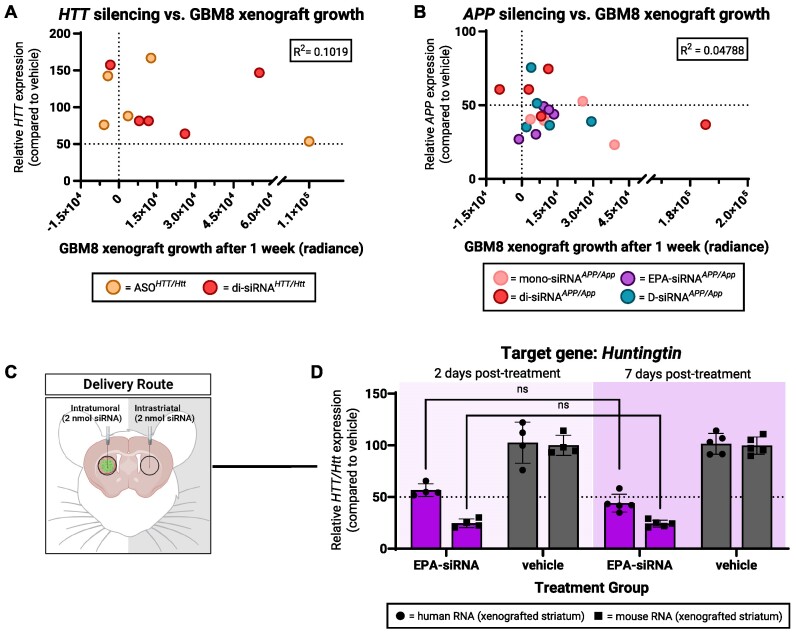
Tumor proliferation does not drive oligonucleotide-mediated silencing patterns in GBM8 xenografts. (A, B) No observed correlation between (**A**) human *HTT* silencing or (**B**) human *APP* and GBM8 xenograft growth following treatment with indicated compounds. (**C**) For bilateral intratumoral and intrastriatal injections of EPA-siRNA, (**D**) comparing human *HTT* silencing at 2 days or 7 days after treatment measured using qPCR. Created with BioRender.com.

Most oligonucleotide-mediated gene silencing in the brain has occurred in post-mitotic tissue where cell division is not a factor. In GBM, tumor cells are rapidly dividing. Continuous cell division would be expected to dilute the effects of the drug more quickly than in a non-proliferative environment, creating challenges for effective drug delivery and cancer treatment. For both *HTT/Htt* (Figure 8A) and *APP/App* studies (Figure 8B), tumor mRNA silencing induced by the ASO or siRNA compounds indicated showed no correlation with GBM8 xenograft growth (calculated using the difference in tumor-associated bioluminescence measured the day before and 1 week after treatment).

To more directly examine whether tumor proliferation accounted for the observed differences in normal brain and GBM gene silencing, we compared tumor *HTT* mRNA levels at 2 and 7 days following intratumoral treatment with EPA-siRNA*^HTT/Htt^*in two separate experiments (Figure 8C). After 2 days, EPA-siRNA*^HTT/Htt^*reduced *HTT* mRNA expression by 44% and after 7 days *HTT* mRNA expression showed sustained reductions at 57% (Figure 8D). The level of *Htt* mRNA in normal mouse cells was reduced by ∼75% at both 2- and 7-days post-treatment with EPA-siRNA*^HTT/Htt^*. Therefore, while tumor proliferation is an important variable and would certainly be expected to affect maintenance of gene silencing long-term, if cell division is unchecked by the therapy, it does not account for the noted differences in GBM silencing at the timepoints used in these studies.

## Discussion

Chemical modifications which minimize toxicity and extend duration of drug activity in brain and spinal cord tissues have expanded the therapeutic applications of oligonucleotides for the treatment of neurological diseases (9). Glioblastoma, however, is characterized by profound and insidious cellular changes that could affect the uptake and efficacy of oligonucleotides that have been chemically optimized for efficacy in other brain diseases.

As such, to study the uptake of different classes of chemically modified and conjugated oligonucleotides in GBM tumors, we developed a qPCR-based assay to differentiate oligonucleotide-induced gene silencing in GBM xenograft cells from silencing in normal brain cells derived from the same tissue sample. We applied this assay across a chemically diverse panel of oligonucleotides to evaluate their activity in patient-derived GBM xenografts and identify compounds that demonstrated effective gene silencing in GBM cells. While the main text figure panels in this paper reflect independent experiments, we have also included a single reference figure summarizing the silencing mediated by the full series of compounds delivered intratumorally to facilitate comparison (Supplementary Figure S5).

In the *HTT/Htt* case, ASOs (Figures 2E, G and 3D) and unconjugated siRNAs (Figure 4C) show weak silencing in tumor cells but induced strong silencing in normal brain cells. In the *APP/App* case, unconjugated siRNAs did show meaningful silencing in the tumor cells, an outcome that diverged from the *HTT/Htt* results—though the silencing in normal brain cells was still generally higher than the silencing in tumor cells (Figure 4D).

Our results suggest there can be meaningful differences in functional oligonucleotide uptake comparing tumor and normal tissue. However, the molecular mechanisms underlying these functional differences are complex and convoluted by tumor heterogeneity. The unconjugated ASO and siRNAs we tested incorporate partial PS-modified backbones (51). In addition to nuclease resistance, PS modifications drive productive oligonucleotide uptake by mediating interactions with trafficking proteins and cell surface scavenger receptors (65). Considering the extent of heterogeneity in patient tumors—which is recapitulated in patient-derived GBM xenograft models (4,66)—it is possible that not every subpopulation of GBM cells (defined by either subtype or cell state) express the genes important for PS-driven uptake. It has been observed that different cell types in complex tissue functionally take up oligonucleotides with varying efficiencies (22,23). Thus, it would not be surprising that similar results could extend to GBM tumors where certain cell subpopulations may be less susceptible to silencing by unconjugated, PS-modified oligonucleotides.

Methods such as single cell RNA sequencing have been invaluable tools for dissecting the nature of complex tissues and will be key for understanding how cell type heterogeneity contributes to efficacy and differential drug responses. In future studies, it will be of great interest to use methods including single cell RNA sequencing to further investigate how the cellular makeup of GBM xenografts and normal brain tissue impacts the distribution of oligonucleotide activity. We are also interested in studying how different chemical designs may change cellular activity profiles (for example, comparing the cell-type specific activity profiles of an unconjugated, PS-modified oligonucleotide to a lipid-conjugated, PS-modified oligonucleotide in xenografts).

### Highly hydrophobic conjugates can be associated with neurotoxicity

We found that siRNAs conjugated to an amphiphilic dendrimer (13,46–48) or the moderately hydrophobic fatty acid EPA (11,43,44) showed better silencing in GBM cells than their unconjugated siRNA counterparts. Previous work showed that cholesterol conjugated siRNAs gave functional silencing in GBM xenografts (19). While the cholesterol conjugated siRNA was also active in our hands, it showed substantially higher neurotoxicity than the EPA and dendrimer conjugated siRNAs we present in this paper. The DCA conjugate we tested was also associated with severe neurotoxicity. We suspect that the neurotoxicity was the result of the very high hydrophobicity from these conjugates, as the overall chemical design of the siRNA itself did not significantly change between compounds. Oligonucleotides with highly hydrophobic conjugates such as cholesterol can disrupt cell membrane integrity and have been associated with *in vivo* toxicity (40,67). Cholesterol-conjugated siRNAs are particularly toxic even in healthy brain and unsaturated fatty acids offer a safer option (67). Generally, in healthy brains, the use of lipid conjugated oligonucleotides is not needed for functional uptake. In cases where lipid conjugation may be needed for effective uptake by specific cell types in the brain, we recommend the use of moderately hydrophobic or amphiphilic lipid conjugates instead of saturated fatty acids or sterols.

EPA is moderately hydrophobic (11) and the dendrimer is amphiphilic (13,48). EPA-siRNA and D-siRNA-treated mice showed recovery patterns comparable to vehicle-injected mice. We did observe a mild seizure-like phenotype in one D-siRNA*^APP/App^* treated mouse during post-operative observation, but the subsequent recovery of this mouse was equivalent to the other mice in the same treatment group which did not exhibit seizures. The seizure was likely sequence-related as two other mice (one from the mono-siRNA*^APP/App^* and one from the di-siRNA*^APP/App^* treated groups) also exhibited mild seizures.

### Effects of tumor xenograft proliferation on oligonucleotide silencing

We assessed the role of tumor xenograft proliferation on oligonucleotide silencing in GBM cells and found that proliferation is not the primary driver for the silencing trends observed. From a quantitative perspective, a single therapeutic oligonucleotide dose typically contains a vast excess of molecules compared to the number of cells. Using the 15 nmol intratumoral ASO dose from our experiment, this translates into >10 million oligonucleotide molecules being delivered per cell near the injection site. In culture, oligonucleotides are also readily delivered to proliferating cell populations over several days. During this time treated cells undergo multiple division cycles but potent RNA silencing (>90%) can still be achieved. In gymnotic delivery—oligonucleotide delivery without the use of a transfection agent—cell division has even been observed in some cases as an advantage which enhances functional uptake (68,69). Nonetheless, we recognize the role tumor proliferation plays in both longer term experiments and in the clinic. We intend to investigate silencing durability of both EPA-siRNA and D-siRNA over extended periods of time and determine at what frequency these compounds would require maintenance dosing.

### Limitations with *in vivo* xenograft cancer models

Patient-derived xenografts are a common and suitable model for studying GBM *in vivo* (70,71). However, the qPCR assay we describe in this work that allows us to differentiate silencing in tumor from silencing in the surrounding tissue is inherently limited to xenograft models. This fact leads to some limitations: for example, it is possible that there might be species-specific differences in RNA trafficking or processing—or even simply differences in RNA structure in the non-conserved regions that have an effect on the accessibility of the target region—making it easier to silence a target in cells from one species than the other. In our work, this risk is mitigated since we compared the same oligonucleotides with different conjugation approaches and made direct and well-controlled comparisons.

The risk is also mitigated by the fact that multiple oligonucleotide classes and mechanisms show higher activity in normal brain cells than tumors. For example, it is striking that both ASOs and di-siRNAs showed higher activity in normal cells than in tumor cells, suggesting that the differences we observe are not simply an artifact of different intracellular localization in the two cell types, but rather reflect differences in PS-backbone-mediated uptake mechanisms between normal brain cells and these GBM xenograft cells.

As a next step in development, our conjugate design insights should be applied to other brain tumor models (syngeneic and genetic models) to ensure applicability in these models as well. While the ability to reliably differentiate silencing in GBM cells vs. normal cells would be more challenging, an impact on tumor progression in these models would provide additional justification for translation toward clinical use.

### Advancing oligonucleotides as neuro-oncology drugs in the clinic

Both nusinersen and tofersen, approved ASO drugs for the treatment of spinal muscular atrophy and SOD1-driven ALS, respectively, are administered directly to the CNS through intrathecal infusions. Conceivably, an oligonucleotide drug for GBM patients could also be administered intrathecally as an adjuvant to surgical resection to mitigate the growth of secondary or recurrent tumors. Indeed, we demonstrated that siRNAs conjugated to amphiphilic and less hydrophobic lipids efficiently distribute to xenografts via CSF flow after ICV injections, a delivery route that parallels intrathecal injections in patients. Alternatively, oligonucleotides could be loaded into an Ommaya reservoir, an implant device that provides direct access to CSF reservoirs and is already used in CNS-related chemotherapy delivery.

Our work underlines that groups interested in developing therapeutic oligonucleotides for GBM cannot always rely on oligonucleotide chemistries that were optimized for normal brain tissue when screening target sequences. Some ASOs or asymmetric siRNAs may be active in tumors without lipid conjugation (as in our *APP/App* example). Similarly, other groups have published ASO approaches with activity in GBM models (20) or other brain cancer models (72). The mechanism underlying these differences in functional uptake between GBM cells versus normal brain cells requires further investigation, but may include differences in cell surface receptor expression, intracellular trafficking, efflux pathways, and other factors. Nevertheless, in all the examples we studied, our compounds showed equal or better activity after EPA or dendrimer conjugation, suggesting that these conjugates might provide a good starting point for groups seeking to achieve gene silencing in brain tumors. Additional studies are required to assess silencing durability of these conjugated siRNAs and evaluate their efficacy when applied to a clinically-relevant GBM target.

## Supplementary Material

gkae260_Supplemental_File

## Data Availability

All data presented in the main text and the Supplementary information are available from the corresponding author upon request.
