# Supplementary material for: Quantifying the activity profile of ASO and siRNA conjugates in glioblastoma xenograft tumors in vivo
Source: Nucleic Acids Res. 2024 Apr 13;52(9):4799–817. doi: 10.1093/nar/gkae260 (PMC11109979; doi:10.1093/nar/gkae260)
Supplement: gkae260_Supplemental_File [file gkae260_supplemental_file.pdf]

SUPPLEMENTARY DATA

Quantifying the activity profile of ASO and siRNA conjugates in glioblastoma xenograft tumors *in vivo*

Samantha L. Sarli[1], Hassan H. Fakih[1], Karen Kelly[1], Gitali Devi[1], Julia M. Rembetsy-Brown[1], Holly R. McEachern[1], Chantal M. Ferguson[1], Dimas Echeverria[1], Jonathan Lee[1], Jacquelyn Sousa[1], Hanadi F. Sleiman[2], Anastasia Khvorova[1,3], Jonathan K. Watts[1,4]

Author Affiliations

[1]RNA Therapeutics Institute, University of Massachusetts Chan Medical School, Worcester, MA, USA
[2]Department of Chemistry, McGill University, Montréal, Québec, Canada
[3]Program in Molecular Medicine, University of Massachusetts Chan Medical School, Worcester, MA, USA
[4]Department of Biochemistry and Molecular Biotechnology, University of Massachusetts Chan Medical School, Worcester, MA, USA

TABLE OF CONTENTS

Supplementary Figure S1
Supplementary Figure S2
Supplementary Figure S3
Supplementary Table S1
Supplementary Table S2
Supplementary Figure S4
Supplementary Figure S5

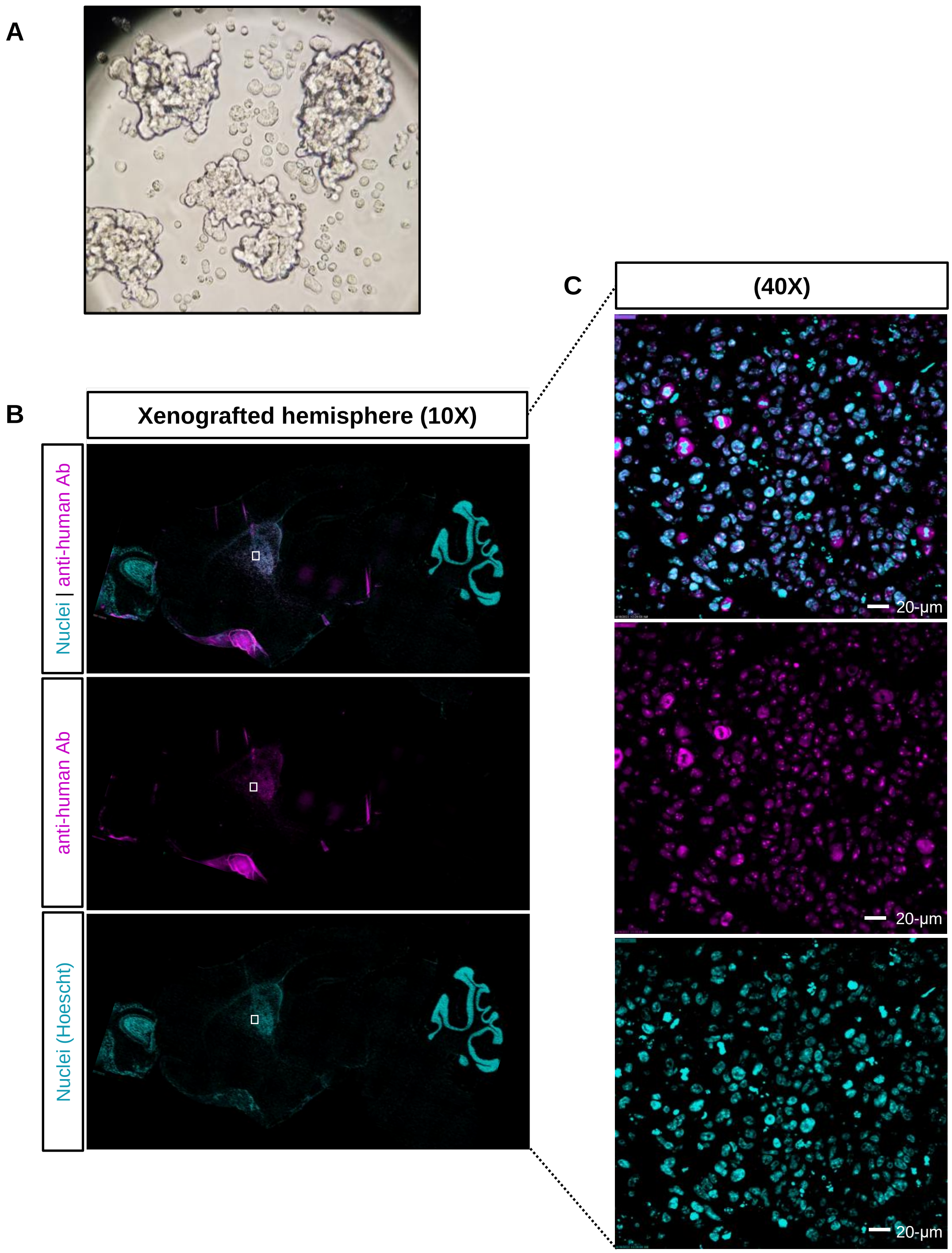

Supplementary Figure S1. (A) Human GBM8 tumorspheres in culture 3 days after thawing from cryopreservation. **(B)** Tiled fluorescent image of sagittal GBM8 xenograft at 4 weeks post-GBM8 implantation and stained with an antibody against human nuclei (anti-human Ab); white squares denote location of **(C)** high resolution image of GBM8 xenograft near tumor core.

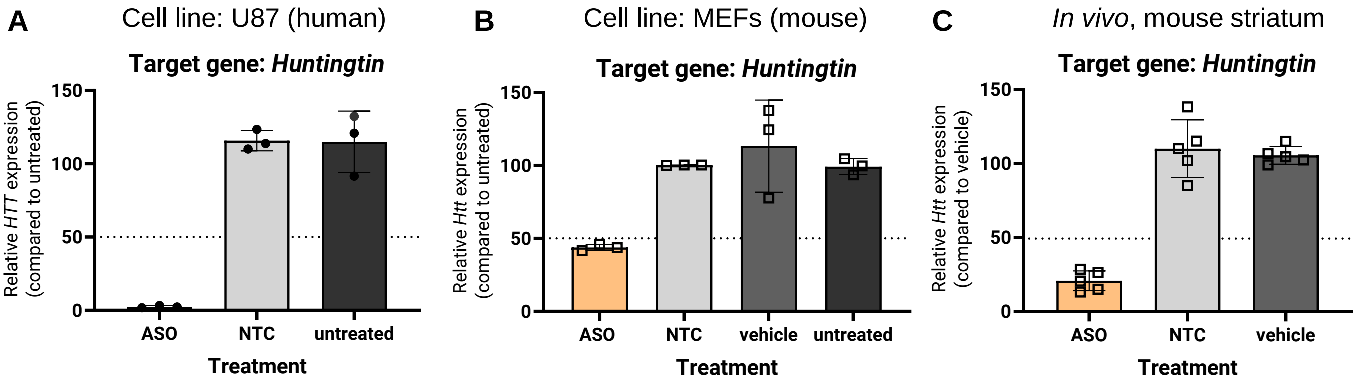

Supplementary Figure S2. Silencing efficacy of homologous *HTT/Htt* ASOs in human and mouse cells. Silencing of **(A)** human *HTT* mRNA in U87 cells or **(B)** mouse *Htt* mRNA in mouse embryonic fibroblasts (MEFs) following lipid transfection (50 nmol, 24-hour treatment). **(C)** Silencing of mouse *Htt* mRNA following intrastriatal injections *in vivo* (15 nmol, 1 week treatment). Each data point represents a biological replicate or individual mouse sample. Data presented are measured using qPCR and treated groups normalized to vehicle or untreated groups.

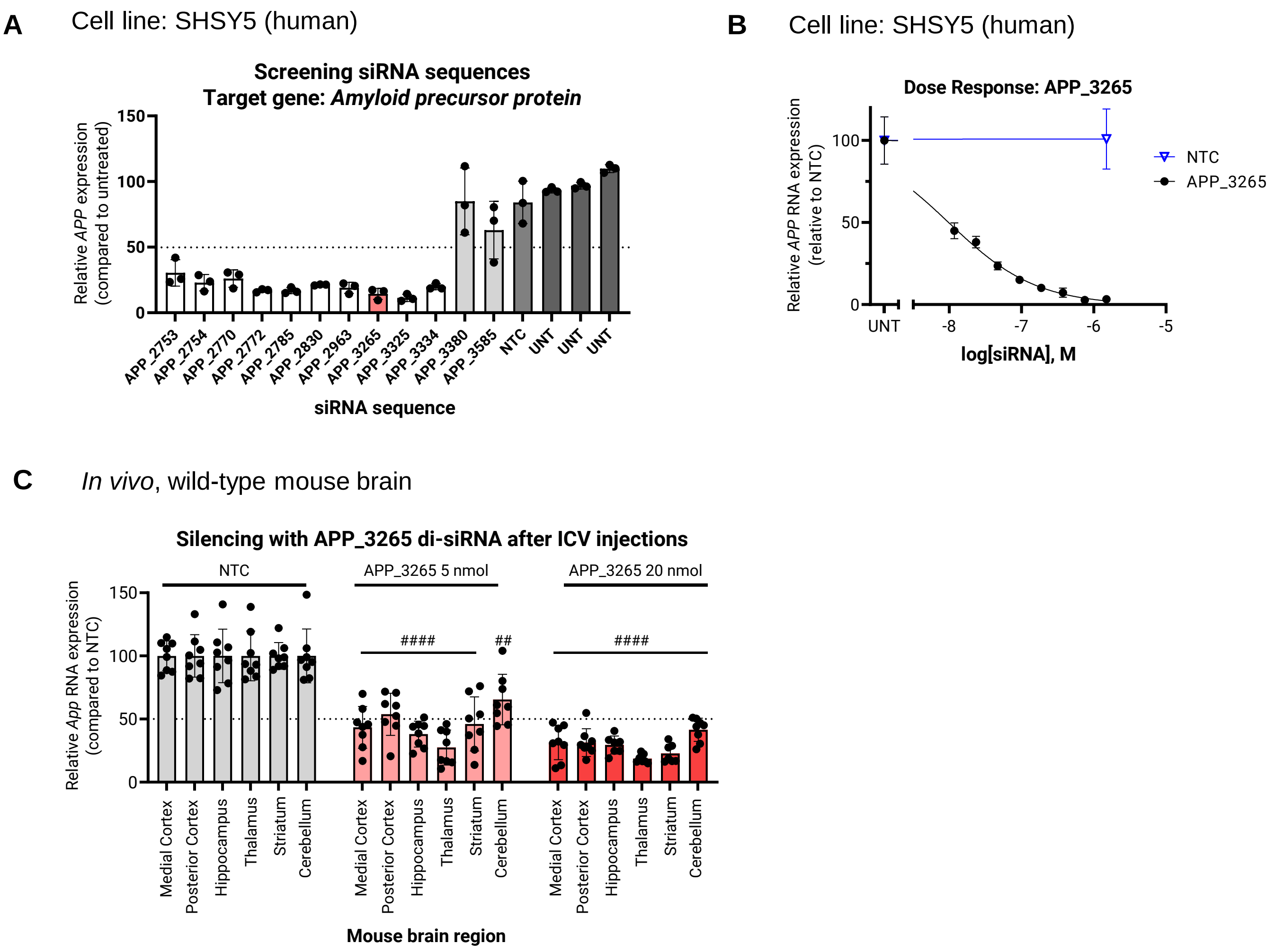

Supplementary Figure S3. Identification and validation of *APP/App* siRNA sequence in human and mouse cells. **(A-B)** Silencing of human *APP* mRNA was measured in SHSY5 cells following cholesterol conjugate-mediated siRNA uptake (1.5 µM, 72-hour treatment): **(A)** identification of lead APP_3265 sequence (in red) from a panel of *APP* targeting siRNAs and **(B)** dose response of lead APP_3265. **(C)** Silencing of mouse *App* mRNA from different brain regions *in vivo,* 1 month following bilateral ICV injections at a total dose of 5 nmol or 20 nmol. Data measured using QuantiGene Assay and treated groups normalized to NTC (non-targeting siRNA control) or UNT (untreated).

Gene	Oligo ID	Sequence (5’-3’)	Accession #
HTT/Htt	ASO	(eC)#(eU)(eC)(eG)(eA)#(dC)#(dT)#(dA)#(dA)#(dA)#(dG)#(dC)#(dA)#(dG)#(dG)#(eA)(eU)(eU)(eU)#(eC)	reference: [24]
	Folate-ASO	Folate(dT)(dT)(dT)(dT)(dT)-<br>(eC)#(eU)(eC)(eG)(eA)#(dC)#(dT)#(dA)#(dA)#(dA)#(dG)#(dC)#(dA)#(dG)#(dG)#(eA)(eU)(eU)(eU)#(eC)	
	siRNA-AS	V(mU)#(fU)#(mA)(fA)(mU)(fC)(mU)(fC)(mU)(fU)(mU)(fA)(mC)#(fU)#(mG)#(fA)#(mU)#(fA)#(mU)#(fA)	NM_002111.6
	di-siRNA-S	(fC)#(mA)#(fG)(mU)(fA)(mA)(fA)(mG)(fA)(mG)(fA)(mU)(fU)#(mA)#(fA)-DIO	
	siRNA-AS	V(mU)#(fU)#(mA)(fA)(fU)(fC)(mU)(fC)(mU)(fU)(mU)(fA)(mC)(fU)#(mG)#(fA)#(mU)#(mA)#(mU)#(mA)#(fA)	NM_002111.6
	Chol-siRNA-S	(mU)#(mC)#(mA)(fG)(mU)(fA)(mA)(fA)(mG)(fA)(mG)(mA)(mU)(fU)#(mA)#(mA)-TegChol	
	DCA-siRNA-S	(mU)#(mC)#(mA)(fG)(mU)(fA)(mA)(fA)(mG)(fA)(mG)(mA)(mU)(fU)#(mA)#(mA)-DCA	
	EPA-siRNA-S	(mU)#(mC)#(mA)(fG)(mU)(fA)(mA)(fA)(mG)(fA)(mG)(mA)(mU)(fU)#(mA)#(mA)-EPA	
	D-siRNA-AS	V(mU)#(fU)#(mA)(fA)(fU)(fC)(mU)(fC)(mU)(fU)(mU)(fA)(mC)(fU)#(mG)#(fA)#(mU)#(mA)#(mU)#(mA)#(fA)	NM_002111.6
	D-siRNA-S	(C12)(SB)(C6)(SB)(dT)(dT)-(mU)#(mC)#(mA)(fG)(mU)(fA)(mA)(fA)(mG)(fA)(mG)(mA)(mU)(fU)#(mA)#(mA)	
	D-siRNA-AS	V(mU)#(fU)#(mA)(fA)(fU)(fC)(mU)(fC)(mU)(fU)(mU)(fA)(mC)(fU)#(mG)#(fA)#(mU)#(mA)#(mU)#(mA)#(fA)	NM_002111.6
	D-siRNA-S	(C12)(SB)(C6)(SB)(dT)(dT)-(mU)#(mC)#(mA)(fG)(mU)(fA)(mA)(fA)(mG)(fA)(mG)(mA)(mU)(fU)#(mA)#(mA)	
APP/App	siRNA-AS	V(mU)#(fG)#(mA)(fG)(fA)(fA)(mU)(fU)(mC)(fU)(mU)(fG)(mG)(fU)#(mA)#(fA)#(mU)#(mU)#(mG)#(fA)#(mU)	NM_001198826.1
	mono-siRNA-S	(mU)#(mU)#(mA)(fC)(mC)(fA)(mA)(fG)(mA)(fA)(mU)(mU)(mC)(fU)#(mC)#(mA)(dT)(dT)	
	EPA-siRNA-S	(mU)#(mU)#(mA)(fC)(mC)(fA)(mA)(fG)(mA)(fA)(mU)(mU)(mC)(fU)#(mC)#(mA)(dT)(dT)-EPA	
	di-siRNA-S	(mU)#(mU)#(mA)(fC)(mC)(fA)(mA)(fG)(mA)(fA)(mU)(mU)(mC)(fU)#(mC)#(mA)(dT)(dT)-DIO	
	D-siRNA-S	(C12)(SB)(C6)(SB)(dT)(dT)(mU)#(mU)#(mA)(fC)(mC)(fA)(mA)(fG)(mA)(fA)(mU)(mU)(mC)(fU)#(mC)#(mA)	
NTC	NTC (ASO)	(eC)#(eC)(eU)(eU)(eC)#(dC)#(dC)#(dT)#(dG)#(dA)#(dA)#(dG)#(dG)#(dT)#(dT)#(eC)(eC)(eU)(eC)#(eC)	reference: [24]
	siRNA-AS	V(mU)#(fA)#(mA)(fU)(mC)(fG)(mU)(fA)(mU)(fU)(mU)(fG)(mU)#(fC)#(mA)#(fA)#(mU)#(fC)#(mA)#(fU)	
	di-siRNA-S	(fU)#(mG)#(fA)(mC)(fA)(mA)(fA)(mU)(fA)(mC)(fG)(mA)(fU)#(mU)#(fA)-DIO	
	siRNA-AS	V(mU)#(fA)#(mA)(fU)(fC)(fG)(mU)(fA)(mU)(fU)(mU)(fG)(mU)(fC)#(mA)#(fA)#(mU)#(mC)#(mA)#(fU)#(mU)	
	EPA-siRNA-S	(mU)#(mU)#(mG)(fA)(mC)(fA)(mA)(fA)(mU)(fA)(mC)(mG)(mA)(fU)#(mU)#(mA)(dT)(dT)-EPA	
	D-siRNA-S	(C12)(SB)(C6)(SB)(dT)(dT)(mU)#(mU)#(mG)(fA)(mC)(fA)(mA)(fA)(mU)(fA)(mC)(mG)(mA)(fU)#(mU)#(mA)	

Supplementary Table 1. Detailed sequences and chemical patterns of oligonucleotides used in this study. Notations are as follows: “#” –phosphorothioate bond, "e" - 2’-*O*-methoxyethyl, "d" - unmodified DNA, "“m” – 2’-*O*-methyl, “f” – 2’-fluoro, 'DIO’ - di-siRNA, “V” – 5’ vinylphosphonate, "Teg" – tetraethylene glycol, "C12" – hexaethylene spacer, "C6" – triethylene spacer, "SB" – symmetrical branching. For siRNAs: “AS” – antisense strand, “S” – sense strand.

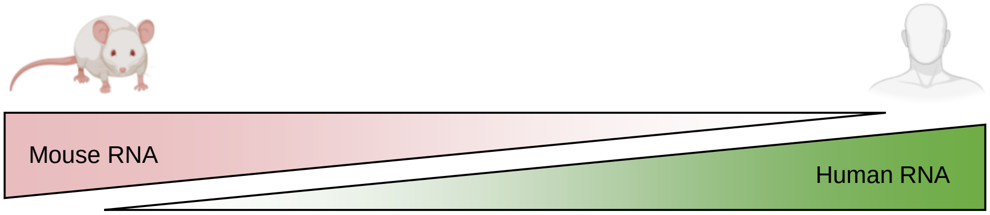

		Ratio of mouse RNA to human RNA								
**Gene**	**Raw Ct**	**100 to 0**	**95 to 5**	**90 to 10**	**70 to 30**	**50 to 50**	**30 to 70**	**10 to 90**	**5 to 95**	**0 to 100**
mouse *Htt*	**Ct1**	27.08	27.24	27.23	27.58	27.72	28.34	29.17	30.13	ND
	**Ct2**	27.00	27.25	27.21	27.32	27.50	28.10	29.29	30.12	ND
human *HTT*	**Ct1**	ND	31.51	30.51	29.24	28.15	27.36	26.96	26.84	26.67
	**Ct2**	ND	31.12	30.48	29.18	28.06	27.32	26.89	26.69	26.75
mouse *Hprt*	**Ct1**	24.51	24.79	24.71	24.93	25.07	25.40	26.44	27.46	ND
	**Ct2**	24.36	24.72	24.53	24.75	24.98	25.44	26.31	27.39	ND
human *HPRT*	**Ct1**	ND	29.07	28.36	27.02	25.58	25.03	24.34	24.09	23.93
	**Ct2**	ND	28.80	28.17	26.98	25.71	24.89	24.37	24.14	23.56
		**100 to 0**	**75 to 25**			**50 to 50**	**25 to 75**			**0 to 100**
mouse *App*	**Ct1**	20.68	21.49			22.05	22.50			ND
	**Ct2**	20.96	21.35			21.90	22.55			ND
human *APP*	**Ct1**	ND	25.37			24.36	23.92			23.99
	**Ct2**	ND	25.08			24.49	23.83			23.60

Supplementary Table 2. Specificity and sensitivity of qPCR probes for human and mouse genes. Pure mouse RNA and human RNA were separately extracted, quantified, and then mixed in different ratios (indicated in table) prior to reverse transcription. qPCR was then performed on the subsequent mixed cDNA samples in technical duplicates (CT1 and CT2). Raw Ct values are reported above. ND = qPCR Ct value > 39 (or non-detectable).

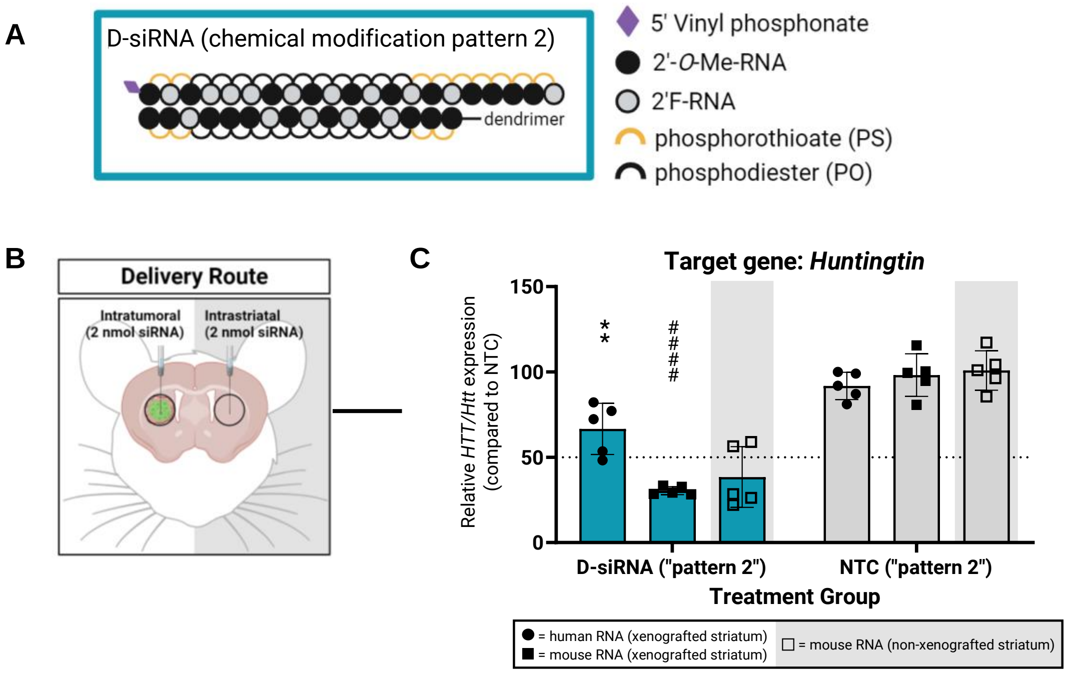

Supplementary Figure S4. (A) D-siRNA chemical modification "pattern 2" siRNA ("dendrimer" refers to (C12)(SB)(C6)(SB)(dT)(dT) as described in Supplemental Table 1). **(B)** For bilateral injections of "pattern 2" D-siRNAs: **(C)** silencing of human *HTT* and mouse *Htt* mRNA measured using qPCR 1 week after treatment.

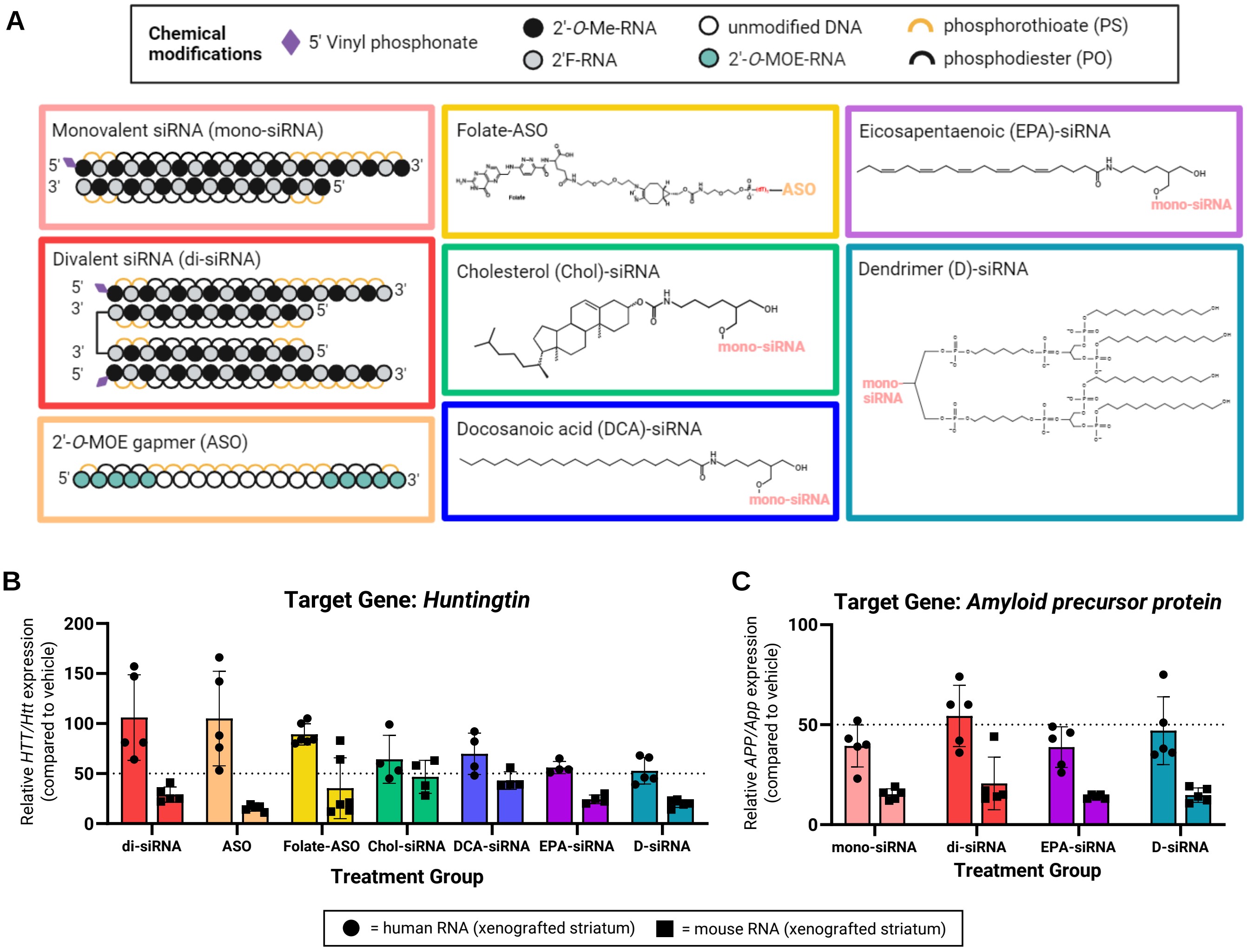

Supplementary Figure S5. (A) Reference summarizing all oligonucleotide compounds delivered to GBM8 xenografts via intratumoral injections across different experiments and their corresponding levels of **(B)** *HTT/Htt* mRNA and **(C)** *APP/App* mRNA silencing.
